# Modelling Representative Population Mobility for COVID-19 Spatial Transmission in South Africa

**DOI:** 10.3389/fdata.2021.718351

**Published:** 2021-10-22

**Authors:** A. Potgieter, I. N. Fabris-Rotelli, Z. Kimmie, N. Dudeni-Tlhone, J. P. Holloway, C. Janse van Rensburg, R. N. Thiede, P. Debba, R. Manjoo-Docrat, N. Abdelatif, S. Khuluse-Makhanya

**Affiliations:** ^1^ Department of Statistics, University of Pretoria, Pretoria, South Africa; ^2^ Foundation of Human Rights, Johannesburg, South Africa; ^3^ Operational Intelligence, NextGen Enterprises and Institutions, Council for Scientific and Industrial Research, Pretoria, South Africa; ^4^ Biostatistics Research Unit, South African Medical Research Council, Cape Town, South Africa; ^5^ Inclusive Smart Settlements and Regions, Smart Places, Council for Scientific and Industrial Research, Pretoria, South Africa; ^6^ Department of Statistics and Actuarial Science, University of Witwatersrand, Johannesburg, South Africa; ^7^ IBM Research, Johannesburg, South Africa; ^8^ College of Graduate Studies, University of South Africa, Johannesburg, South Africa

**Keywords:** COVID-19, spatial, mobility, spatial weight matrices, principal component analysis, hierarchical clustering

## Abstract

The COVID-19 pandemic starting in the first half of 2020 has changed the lives of everyone across the world. Reduced mobility was essential due to it being the largest impact possible against the spread of the little understood SARS-CoV-2 virus. To understand the spread, a comprehension of human mobility patterns is needed. The use of mobility data in modelling is thus essential to capture the intrinsic spread through the population. It is necessary to determine to what extent mobility data sources convey the same message of mobility within a region. This paper compares different mobility data sources by constructing spatial weight matrices at a variety of spatial resolutions and further compares the results through hierarchical clustering. We consider four methods for constructing spatial weight matrices representing mobility between spatial units, taking into account distance between spatial units as well as spatial covariates. This provides insight for the user into which data provides what type of information and in what situations a particular data source is most useful.

## 1 Introduction

The COVID-19 pandemic starting in the first half of 2020 has changed the lives of everyone across the world. From working from home at all hours, using less public and personal transport, home-schooling under lock down, to economic strife and anxiety; predicting such changes would have been near impossible a priori. By far the largest impact, aside from the economic troubles many find themselves in, is reduced mobility. Daily commuting has been much reduced due to various lockdown measures internationally. In addition, international flights and cross border travel was restricted for significant periods of time, even between regions in some countries.

Reduced mobility was essential, however, due to it being the largest impact possible against the spread of the little understood SARS-CoV-2 virus. Social distancing and stay at home instructions were understood and implemented internationally. These instructions were seen as the best protection for the individual, as well as being the means to prevent overload on the hospital systems, which would otherwise result in inflated death rates. These protection mechanisms are formed on an understanding of the basic nature of the spatial spread of the virus. A virus spreads via a host, whom it relies on to move amongst other susceptibles. The more movement and interaction performed by the host, the more the virus is able to spread. It is thus imperative to incorporate a spatial element when modelling the spread of the COVID-19 pandemic. Herein, we focus on modelling the mobility spatially.

Quantifying mobility patterns of people facilitates a more accurate understanding of the spread of the disease. An individual’s ability to physically “lock down” and stay at home was affected by economic inequality, as shown in a US study (Huang et al., 2021). In South Africa, this economic inequality is extreme, with the World Bank recognising South Africa, in 2019, as having the worst inequality in the world^1^.

While the strict lockdown introduced by the South African government from March 27, 2020 delayed the first wave, the mobility was by no means completely reduced due to many living day-to-day for food. Food parcel queues from food donations were a large focus during the first half of the pandemic in South Africa, as the risk of contracting COVID-19 was overridden by the need for food. Such queues, and the use of public transport during these times, heightened the transmission risk of COVID-19 in South Africa, even while lockdown rules were in place. A full lockdown was therefore not possible, and spatial interaction continued between individuals from different regions across South Africa. Modelling regions in isolation will therefore not capture the influence of this mobility on the spread of COVID-19 in South Africa. The use of mobility data in modelling COVID-19 is thus essential to capture the intrinsic spread through the population. A common source is mobile phone location data, which has been utilized previously for epidemiological modelling (Cummings et al., 2004; Wesolowski et al., 2012; Bengtsson et al., 2015; Wesolowski et al., 2015; Finger et al., 2016; Ruktanonchai et al., 2016). However, this data is difficult to obtain due to increasing privacy concerns worldwide. In addition, there are often a number of network providers in a region, each with certain market share. Without data access from all, or at least, the largest providers, representativeness and mobile phone penetration will be limited and should be used with caution. Other sources of mobility data are published by Facebook and Google. The spatial resolution of these is lower, however. In this paper we focus on mobile phone and Facebook mobility data, which has higher spatial resolution than the Google alternative.

It is necessary to determine to what extent different sources of mobility data, at differing spatial resolutions, convey the same message of mobility within a region. In this paper we demonstrate, through the use of principal component analysis as well as hierarchical clustering, how different sources of spatial mobility data at various resolutions can lead to different conclusions with regards to spatial unit connectivity. Spatial connectivity is an essential first step in spatial modeling, providing a quantification of the spatial dependency between spatial units. Herein, we compare the calculation of a number of spatial weight matrices in quantifying how spatial units relate. We also discuss the advantages of different sources and how they can be harnessed when modelling the spread of a virus. We do this by using principal component analysis in order to condense the information that can be gained from a spatial weight matrix and then using hierarchical clustering to identify the strongest spatial associations and to essentially put on display what type of relationships the spatial weight matrix is identifying. This is to the best of our knowledge the first time this exact combination has been used for this purpose.

The mobility data available for South Africa is presented in Section 2. The methodology for constructing connectivity matrices is developed in Section 3, and the results are presented in Section 4. Section 5 provides a discussion and Section 6 concludes.

## 2 Data

Available mobility data is at different resolutions. For the case of South Africa, the administrative divisions of the country are summarised in Table 1. In order of increasing spatial resolution these are country, province, district municipality, local municipality, and ward, labelled as administrative levels 0 through 4 respectively. To facilitate the comparison of different sources of spatial information, it is first necessary to aggregate the data from each source to the same spatial resolution. Increasing the resolution of spatial data can be achieved through methods such as small area estimation or spatial micro-simulation (see e.g. (Ballas et al., 2005; Pfeffermann, 2013)). These methods are somewhat involved and require the use of auxiliary information or assumptions that are unlikely to be true. In this paper we investigate aggregating down to the lowest spatial resolution used by our data sources. While this is relatively straightforward to accomplish, it potentially results in the loss of information.

**TABLE 1:** South Africa’s administrative boundaries.

Administrative level	Spatial unit name	Number of spatial units
0	Country	1
1	Province	9
2	District municipality	52
3	Local municipality	213
4	Ward	4,392

Mobility data are used to understand various issues ranging from epidemic modelling, transport planning and management, communication network improvement and urban planning (Asgari et al., 2013; Zhou et al., 2018). Asgari et al. (2013) indicates that mobility goes far beyond mere geographical movement of humans, but provides a comprehensive perspective on human interactions that could be considered from spatial, temporal, and contextual aspects. Human mobility is one of the aspects of mobility that gained attention from the global spread of infectious diseases as with the recent COVID-19 pandemic. A variety of technologies including navigation sensors, wireless technologies, and cellular communication technologies are used to position humans in space (Toch et al., 2019). A study by Zhou et al. (2018) provides a comprehensive overview of the different types of human mobility patterns data. These include those data types that capture both the wider (city-wide) and minute (building-wide or large structure) human movements, for example, cellular services records (CSRs), surrounding WiFi access point records (SWAPRs), Global Positioning System locations (GPSLs), geotagged social media (GTSM), public transport smart card records (PTSCRs), bluetooth detection records (BDRs), and WiFi probe request records (WFPRs). The analysis methods range from data visualisation to statistical analysis methods (classification and clustering), heuristic logic, graph theory and optimization techniques.

### 2.1 South Africa’s Lockdown Levels

To quell the spread and impact of the COVID-19 pandemic, the South African government instigated one of the strictest lockdowns in the world. This particular lockdown strategy is structured around different “levels” of lockdown, each of which brings different restrictions (with level 5 being the highest and placing restrictions on nearly all forms of travel to all citizens except for those classified as essential workers). The various levels as well as the dates for which they were active are given in Table 2. Note that for this paper we only consider the lockdown until the end of Level 3 due to data availability only over this period.

**TABLE 2:** South Africa’s lockdown levels and dates.

Level	Date	Restrictions
Business as usual	March 1, 2020–March 26, 2020	No restrictions
Level 5	March 27, 2020–April 30, 2020	Essential services only otherwise all confined to place of residence. No inter-provincial movement, except for transportation of goods and exceptional circumstances e.g. funerals. Public and private transport restricted to certain times of the day with limitations on vehicle capacity
Level 4	May 1, 2020–May 31, 2020	More sectors permitted with restrictions, including mining, and partial e-Commerce allowed. Public places (such as religious, cultural, recreational facilities) and the tourism sector remain closed and gatherings prohibited. All confined to place of residence from 8pm to 5am. No local (between metropolitan areas or districts) or inter-provincial movement of people, except for permitted reasons e.g. returning for alert level 4 operations. All borders remain closed except for designated ports of entry for restricted home affairs operations and for the transportation of fuel, cargo and goods. Public and private transport may operate at all times of the day, with limitations on vehicle capacity
Level 3	June 1, 2020–August 17, 2020	More sectors permitted including take away restaurants, e-commerce and delivery services and global business services. Public places and tourism opened and gatherings and sporting activities permitted but all subject to restrictions. All confined to place of residence from 11pm to 4am. No inter-provincial movement of people, except for transportation of goods, exceptional circumstances and other permitted reasons. Public and private transport may operate at all times of the day, with limitations on vehicle capacity

As non-pharmaceutical interventions (such as the lockdown) are eased the population is allowed to become more mobile. Naturally this will have an impact on the transmission rate of the virus and thus this temporal element must be included in some manner. In this paper we split the data temporally on the date ranges given in Table 2 up to level 4 and set up a spatial weight matrix for each level of lockdown to study how mobility patterns changed. Two mobility data types were available for this research. The first is freely available data shared by Facebook, and the second is mobility data made available by a South African cellular provider for the context of the COVID-19 response in 2020. In Figure 7 we provide the Google mobility data at country level. We do not use this data in this research as it is only available at administrative level 1, representing low spatial resolution. It is however useful for context providing mobility levels in each various industry sectors. Mobility for residential travel (i.e., individuals remaining at their place of residence) is the only type of travel that saw an increase after the country transitioned into level 5. Grocery and pharmacy travel saw an initial spike shortly before the country went into level 5 (possibly attributed to panic-buying). After transitioning to level 5 we see a drastic decrease in all types of travel, with residential travel showcases a slightly downward trend while all other forms of travel have an upward trend. Grocery and pharmacy travel is the quickest to recover to pre-COVID levels while travel to parks and travel stations is the slowest to recover (most likely due to this being for leisure). By the end of the year residential travel is still higher than before any lockdown interventions. Table 3 provides the average changes over each level as well.

**TABLE 3:** Average changes in population mobility over lockdown levels using the Google mobility data during 2020.

Level	Date	Retail	Grocery and pharmacy	Parks	Transit stations	Workplaces	Residential
BAU	2 Feb - 26 Mar	−3.49	1.68	−9.39	−5	−0.88	1.71
Level 5	27 Mar - 30 Apr	−73.06	−46.09	−46.86	−78.49	−65.89	33
Level 4	1 May - 31 May	−50.39	−23.45	−39.39	−61.71	−40.58	23.35
Level 3	1 Jun - 17 Aug	−29.53	−10.71	−23.17	−49.72	−28.1	17.17
Level 2	18 Aug onwards	−17.76	−3.34	−23.29	−34.65	−19.78	11.35

### 2.2 Facebook Data for Good

Multiple geographically indexed datasets have been made freely available for use by Facebook through their “Facebook data for good” initiative. These datasets serve to aid researchers and policymakers in understanding the spread of COVID-19^2^.

This paper utilises one of these available datasets, namely the “Movement range maps” dataset. The data indicates the change in mobility, $F_{i}^{\left(t\right)}\in \left(-1,1\right)$ (which can be interpreted as a percentage (−100, 100)), for a spatial unit *i* on a given day *t* over the period March 1, 2020–February 28, 2021 relative to a 1-week baseline calculated in February 2020. The daily values for each district municipality were calculated by determining the number of so-called “Bing tiles”^3^ that each inhabitant visited on a given day (place of residence being determined by the location where users most often spend their nights). A bing tile is the term used by Microsoft for a spatial polygon. After incorporating some degree of noise, the average number of tiles visited by the inhabitants was determined and expressed relative to the baseline. The full description of how these values were calculated is available in the Appendix. The spatial resolution for units of this data are district municipalities, namely at administrative level 2.

**FIGURE 1:**
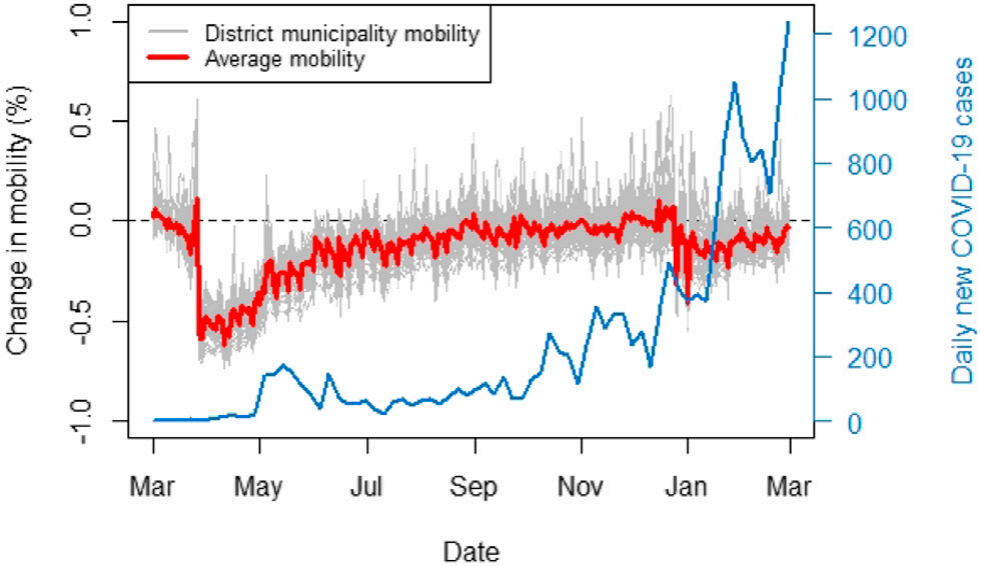
“Facebook for good” movement range maps data (March 1, 2020–February 28, 2021) relative to a baseline calculated in a week of February 2020.

Figure 1 shows the aggregated data for district municipalities, with the average across the district municipalities shown in red. The figure demonstrates that the average mobility nationally dropped significantly in late March. This corresponds to when South Africa entered its first hard lockdown on the March 27, 2020 (see Table 2). The hard lockdown imposed severe restrictions on travel and constituted a strict stay at home directive. Only essential workers were allowed to leave their homes. Furthermore, the average change in mobility is primarily negative over the entire study period, indicating that mobility patterns remain more constrained than before the hard lockdown. The first COVID-19 case was discovered on March 5, 2020 and the lockdown announcement was made a week later on 15 March. This could explain the drop in mobility already seen from early March.

Notable benefits of using this data are that the data is freely available and could potentially act as a very representative proxy for human mobility, as Facebook services are not constrained to specific mobile network providers. In addition, all the cellular network providers in South Africa provide a free version of Facebook called Facebook Zero. Even though it is known that not all South Africans have a Facebook account, the Facebook mobility data may provide an acceptable level of representativeness for mobility within the country since the population of South Africa is considered significantly young^4^. It is also clear that a large amount of the original data was censored in order to preserve user privacy and thus the data is at a sparse level of spatial resolution (administrative level 2). The data is also not specific with regards to the direction of spatial mobility. Daily observations only indicate whether individuals were more or less mobile in a district municipality and do not indicate the spatial units towards which this mobility was directed.

### 2.3 Mobile Network Data

The growing popularity and widespread use of mobile devices has led to massive amounts of data being produced at any given point in time all around the world. Mobile phone data can be collected either passively by mobile services providers or through the use of mobile applications. The ease with which such large quantities of data can be gathered makes cellular data attractive for researchers. Mobile devices operate by sending and receiving information from cellphone towers. When interacting with a cellphone tower we say that a phone has “pinged” off a cellphone tower. A mobile device may ping off a cellphone tower by sending or receiving any kind of information, be it a phone call, text message or application notification. The mobile network data obtained for this research is obtained using the number of users whose mobile devices pinged off a cellphone tower within one ward (administrative level 4) on a given day and then later that day pinged off a cellphone tower in a different ward.

Mobile phone data has been used numerous times in the field of spatial epidemiology to model the spread of various diseases, including cholera (Bengtsson et al., 2015; Finger et al., 2016), dengue (Cummings et al., 2004; Wesolowski et al., 2015) and malaria (Wesolowski et al., 2012; Ruktanonchai et al., 2016). Following the outbreak of the COVID-19 pandemic, the governments of various countries across the world began collecting cellular device user data in an attempt to aid the conception and implementation of non-pharmaceutical interventions (Ekong et al., 2020; Oliver et al., 2020; Peixoto et al., 2020; Varsavsky et al., 2021). This data has since been used by researchers to clearly establish a correlation between population mobility and COVID-19 case numbers (Gao et al., 2020; Peixoto et al., 2020; Xiong et al., 2020; Zhou et al., 2020).

Limitations of mobile phone data exist. First and foremost of these is the issue of user privacy. Mobile phone data could potentially be misused to identify specific individuals and thus cellular providers are often hesitant to provide researchers with such data (Grantz et al., 2020; Oliver et al., 2020). Such data is often aggregated to a low spatial resolution to prevent this as well as reduce noise, but this comes at the cost of some data specificity. Another potential drawback of mobile phone data is high computational cost. For high mobile phone penetration rates, mobile phone data may consist of a number of entries in the order of billions. The computational cost of processing such datasets is prohibitive, potentially preventing analysis.

For this paper, anonymised mobile phone data was obtained from a local mobile network provider. In South Africa, the mobile phone penetration level is estimated to be as high as 95%^5^. The provider utilised in this paper is one of the largest providers in the country, with an estimated market share of 42%.

**FIGURE 2:**
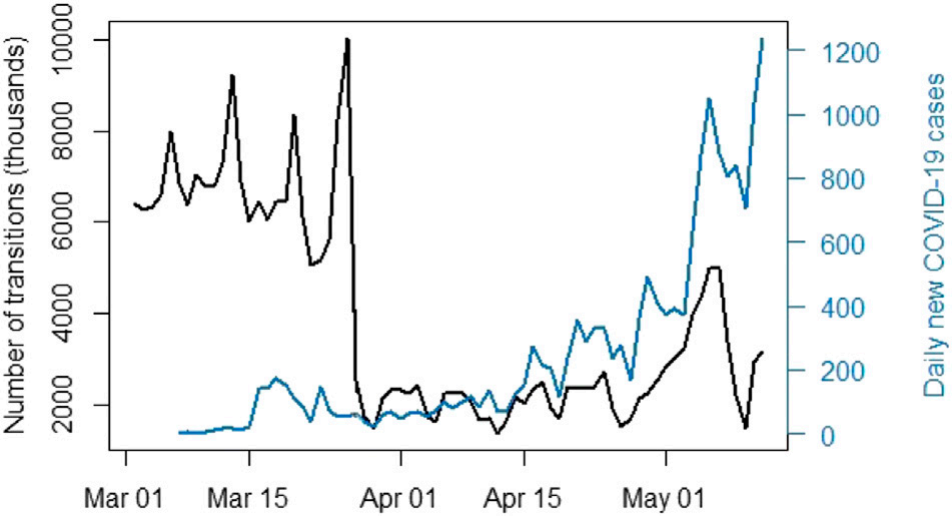
Number of individual transitions between wards using the available mobile phone data (March 2, 2020–May 12, 2020).

**FIGURE 3:**
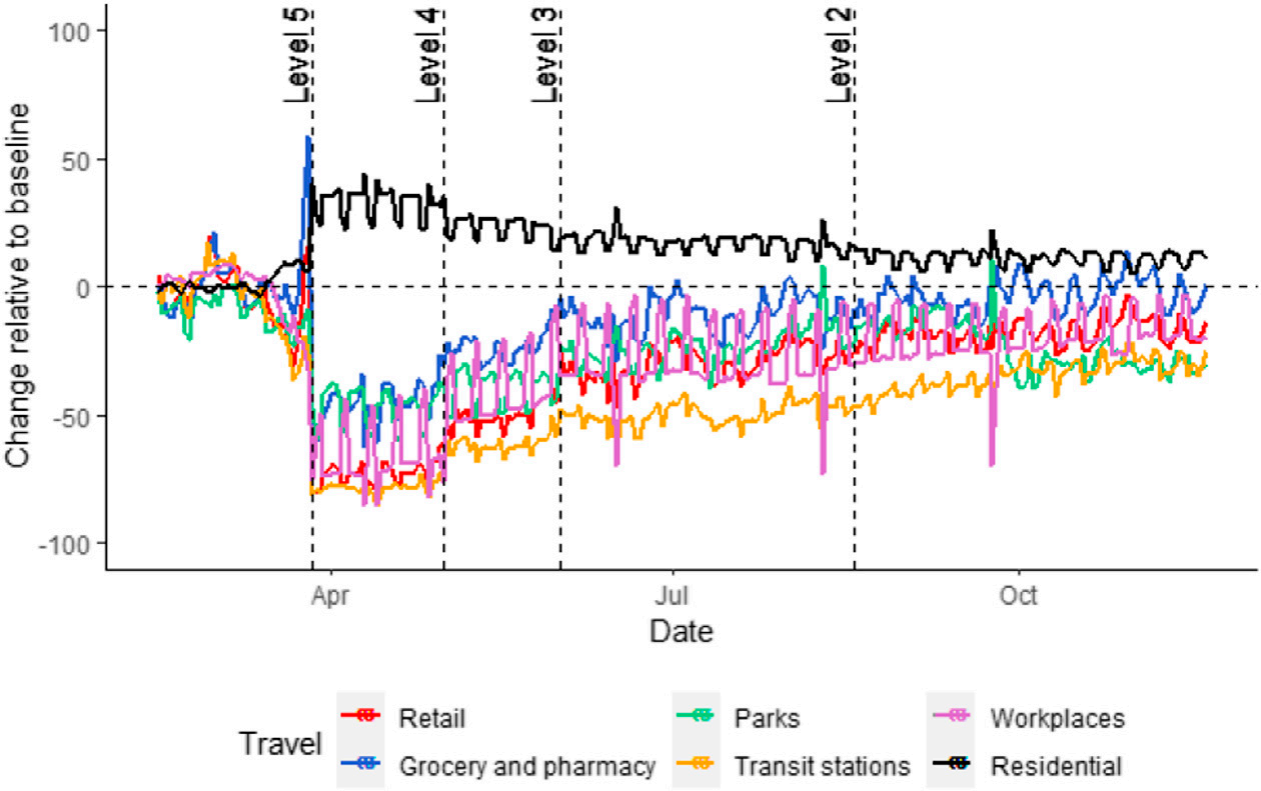
Google mobility report data for February 15, 2020–November 20, 2020 (transitions to different levels of lockdown indicated by vertical reference lines).

The data provides the number of mobile phone users $m_{ij}^{\left(t\right)}$ that travelled to ward *j* from ward *i* on day *t* for the period 2 March - May 12, 2020. The data is at administrative level 4, which is the highest spatial resolution reasonably possible while preserving some level of privacy of exact user location. To compare insights gained from this data and the Facebook dataset in Section 2.2, it would first be necessary to aggregate the mobile phone data to the same spatial resolution which is administrative level 2. In South Africa, each ward has a unique 8-digit ID code. The first three digits of this code indicates the district municipality that the ward is a part of. For example, the ward ID 9344007 indicates that the ward is part of the district municipality with code 934. In order to aggregate the data to district municipality level, one could replace the ward IDs of the observations with their district municipality codes (i.e. only the first three digits), whereupon rows with identical origin and destination codes would be discarded. The mobile phone data at administrative level 2 is thus given by

$$M_{I,J}^{\left(t\right)}=\underset{i\in I,j\in J}{\sum }m_{ij}^{\left(t\right)},$$

where *I* and *J* are district municipalities and *i* and *j* are wards as previously indicated. Transitions contained within a single district municipality are thus discarded. Analysis revealed that this caused an average of 26% of daily observations to be discarded. The retained data is displayed in Figure 2. The representation differs to that of Figure 3 as the data provides transitions between regions in this case. We once again notice a sharp decline in population mobility in late March.

The population of South Africa (mid-2021) is approximately 60.14 million^6^, and yet the highest total number of inter-district municipality transitions on any given day was approximately 10 million (seen in Figure 2). It should be noted that the same individual can be responsible for multiple transitions and that some individuals could potentially possess multiple cellular devices. Literature does exist on the use of mobile phone data to estimate population numbers, see e.g. (Sakarovitch et al., 2018). Doing so is not within the scope of the research presented here but would be of value in testing mobile phone representability. Despite the quality of available hardware^7^, this process proved highly computationally expensive due to the number of comparisons that need to be run on billions of lines of data in order to create a spatial weight matrix for each day in the time period.

## 3 Methodology

### 3.1 Literature Review

When a particular phenomenon exhibits evidence of spatial dependence, this dependency must be taken into account when modelling to minimise the risk of producing biased results (Stakhovych and Bijmolt, 2009; Ejigu and Wencheko, 2020). In the case of an infectious disease that is spread through physical contact and near proximity, it is clear that locations that are situated closer together (or rather the inhabitants of these locations) will play a larger role in determining their respective infection rates than locations that are farther apart. To incorporate this fact, spatial models allow spatial units to be more strongly (or weakly) correlated with one another based on some select criteria that is deemed suitable for the phenomenon being modelled. This is achieved through the use of a spatial weight matrix (sometimes called a “spatial mobility matrix”) usually denoted by ***W*** (Bavaud, 1998; Getis and Aldstadt, 2004; Aldstadt and Getis, 2006; Stakhovych and Bijmolt, 2009; Anselin, 2013; Ejigu and Wencheko, 2020).

**Definition 1** (Spatial weight matrix). Let *S* = {1, 2, *…* , *n*} be a set of spatial units. A spatial weight matrix (Bavaud, 1998; Getis and Aldstadt, 2004; Stakhovych and Bijmolt, 2009; Anselin, 2013) is an *n* × *n* matrix ***W*** = [*w*_*ij*_] satisfying *w*_*ij*_ ≥ 0 and $\sum_{j=1}^{n}w_{ij}=1\;\forall \;i\in S$ .

This matrix is formally defined as an expression of spatial dependency between spatial units (Bavaud, 1998; Getis and Aldstadt, 2004; Stakhovych and Bijmolt, 2009; Anselin, 2013). Simply put, the spatial weight matrix is constructed in such a way so that entry *w*_*ij*_ quantifies the amount of spatial influence that spatial unit *i* exerts on spatial unit *j* (Bavaud, 1998; Getis and Aldstadt, 2004; Stakhovych and Bijmolt, 2009; Anselin, 2013).

Such matrices are frequently restricted to being symmetrical to simplify estimation. However, symmetry is not required and can result in a less realistic representation of spatial dependency (Bavaud, 1998; Getis and Aldstadt, 2004; Stakhovych and Bijmolt, 2009; Anselin, 2013). Another common convention is that *w*_*ii*_ = 0 for all *i* to exclude the possibility of so-called “self-influence” (Bavaud, 1998; Getis and Aldstadt, 2004; Stakhovych and Bijmolt, 2009). Non-zero diagonal entries can however be included and are interpreted as quantifying the resistance that each spatial unit has against influence from the other spatial units (Bavaud, 1998; Anselin, 2013). Performing row-standardisation on the matrix allows the connectivity of different spatial units to be compared (Bavaud, 1998; Getis and Aldstadt, 2004).

Spatial weight matrices are most commonly used in the fields of econometrics and spatial statistics (Anselin, 2013). Recently however, they have become popular in the field of spatial epidemiology and have been used to model various diseases including dengue, malaria, foot and mouth disease (Brown et al., 2016; Malik et al., 2016; Brown et al., 2018; Suryowati et al., 2018) and most recently COVID-19 (Huang et al., 2020; Tagliazucchi et al., 2020). There are relatively few established guidelines with regards to constructing a spatial weight matrix (Bavaud, 1998; Aldstadt and Getis, 2006; Stakhovych and Bijmolt, 2009; Ejigu and Wencheko, 2020), however, the construction of these matrices has seen some advancement, with greater emphasis being placed on creating matrices that offer an accurate representation of human mobility. Simpler models rely on measures such as distance, contiguity or adjacency (Aldstadt and Getis, 2006; Stakhovych and Bijmolt, 2009; Anselin, 2013; Brown et al., 2016; Malik et al., 2016; Brown et al., 2018; Suryowati et al., 2018; Ejigu and Wencheko, 2020; Huang et al., 2020) while more complex ones are able to use mobile phone data (Huang et al., 2020) and geostatistical information (Getis and Aldstadt, 2004; Aldstadt and Getis, 2006). Accurately specifying these matrices is a non-trivial problem, as discussed in (Ejigu and Wencheko, 2020). Most recently, Ejigu et al. proposed a methodology through which both distance and covariate information can be utilized (Ejigu and Wencheko, 2020).

Given the importance of correctly specifying the spatial weight matrix, and the fact that there are often multiple sources of spatial data available on hand, it becomes necessary to develop some means of comparing spatial weight matrices. Specifically, it is necessary to compare the insights that can be derived from different spatial weight matrix definitions. In recent years this comparison has been achieved either through the use of measures of spatial autocorrelation, such as Moran’s I (Suryowati et al., 2018), or through more specialised methods local to the field of spatial statistics (Gao et al., 2018; Jin et al., 2020). In this paper, we adapt an idea initially presented by Garrison and Marble (Garrison and Marble, 1964), whereby principal component analysis is used to reduce the dimensionality of candidate spatial weight matrices. We then introduce the use of hierarchical clustering to derive a clustering solution for the spatial unit principal scores. This allows for a more informative comparison of the information provided by these connectivity matrices, as opposed to simply comparing their structure visually.

### 3.2 Spatial weight Matrices

Selecting an optimal spatial weight matrix is often reliant on the use of a priori information and experience. In this paper the emphasis is on comparing the implications for different spatial weight matrices and the varying types of spatial associations that they represent. We next discuss the spatial weight matrix construction approaches used in this paper.

#### 3.2.1 Method 1: Distance Method

The exponential distance definition of a spatial mobility matrix is used frequently in studies involving spatial correlation, and is a popular choice in spatial econometrics (Aldstadt and Getis, 2006; Stakhovych and Bijmolt, 2009; Anselin, 2013; Ejigu and Wencheko, 2020). As previously mentioned however, the concepts of distance, contiguity and adjacency do not necessarily offer the most accurate or realistic representation of human mobility. In this paper we include this model in order to draw comparisons between it and more data-driven models. The entries of the spatial weight matrix are given by

$$w_{ij}=\exp\left(-d_{ij}\right)$$ (1)

where *d*_*ij*_ is the Euclidean distance between the centroids of spatial units *i* and *j*. Diagonal entries are set to 0 to remove the possibility of so-called “self-influence,” and all rows are standardised to sum to 1 to facilitate comparisons between different spatial units. Both of these restrictions were maintained for all matrices in this paper. Under this model, spatial units are most strongly spatially correlated with the spatial units that are closest to them geographically. No temporal component can be incorporated for this method.

#### 3.2.2 Method 2: Mobile Network Method

The mobile network data indicates the number of individuals that travelled from spatial unit *i* to spatial unit *j* on a given day *t*. These entries are used to construct the spatial weight matrix as follows,

$$w_{ij}^{\left(t\right)}=M_{ij}^{\left(t\right)}.$$ (2)

This model expresses spatial weights as a function of the amount of flux (both in and out) occurring at a spatial location, and is sometimes referred to as a spatial interaction matrix (Bavaud, 1998). Spatial units where more (less) individuals travelled to other spatial units will thus have a larger (smaller) effect on other spatial units.

#### 3.2.3 Method 3: Weighted Facebook Data Method

In order to create a spatial mobility matrix using the Facebook data, we use the same approach of Ejigu et al. (Ejigu and Wencheko, 2020). This takes into account proximity as well as covariate information which is spatially dependent. The entries of the spatial weight matrix are given by

$$w_{ij}^{\left(t\right)}=\exp\left(-\left(\alpha \cdot |F_{i}^{\left(t\right)}-F_{j}^{\left(t\right)}|+\left(1-\alpha \right)\cdot d_{ij}\right)\right)$$ (3)

where $F_{i}^{\left(t\right)}$ is the mobility of spatial unit *i* at time *t*, scaled by population size (the covariate information), *d*_*ij*_ is the Euclidean distance between the centroids of spatial units *i* and *j*, and *α* ∈ (0, 1) is a control parameter indicating the amount of weight that should be given to the covariate term. The control parameter *α* was set to 0.6 in this paper to allow for the covariate data to play a slightly more prominent role in the estimation process without disregarding the importance of distance. The parameter captures that we are making an assumption that the Facebook data can be used to capture transitions between regions even though it is isolated location data. The value of 0.6 gives the weighted calculation a slight nudge towards the Facebook data. Note that if *α* = 0 then the model simplifies to the exponential distance model in Eq. 1.The Facebook mobility data for each district municipality was scaled using population size in order to account for the fact that increased mobility in a given district is more (less) influential to neighbouring districts if the population size is large (small). This was also done in order to restore some of the variation in the data that was likely lost when the data was censored to a lower spatial resolution.

#### 3.2.4 Method 4: Scaled Facebook Data Method

An additional final spatial weight matrix was constructed based on further variation of the exponential distance model. For this matrix, the rows of the exponential distance matrix are scaled using the (unscaled) Facebook mobility data. For example, if the mobility within district municipality *i* was 20% lower than the baseline, then the entire row *i* is multiplied by 0.8. Each entry in the exponential distance matrix is thus scaled by some number in (0,2). The entries in the matrix are given by

$$w_{ij}^{\left(t\right)}=\left(1+F_{i}^{\left(t\right)}\right)\cdot \exp\left(-d_{ij}\right).$$ (4)

This construction allows the exponential distance matrix to be scaled such that the spatial influence of more (less) mobile district municipalities is increased (decreased). This also renders the exponential distance matrix non-symmetric, which should offer a more realistic representation of spatial influence. Methods three and four are a novel approach to constructing connectivity matrices from the Facebook mobility data.

### 3.3 Principal Component Analysis

Principal component analysis (PCA) is a statistical technique that aims to derive a parsimonious representation of a given dataset by deriving an orthogonal linear transformation of the data (Friedman et al., 2001). In standard PCA, the only hyperparameter that needs to be selected is the number of principal components, which is primarily dependent on the cumulative proportion of variance in the data that the user wishes to retain. For this paper, the number of principal components was chosen such that 75% of the variation in the data was maintained. The full discussion of PCA and its various extensions is left to the existing literature (see e.g. (Friedman et al., 2001)).

### 3.4 Hierarchical Clustering

Hierarchical clustering is an unsupervised machine learning technique that allows the user to group together data points in an attempt to uncover sets of observations that share similar characteristics (Friedman et al., 2001). This is achieved by procedurally grouping together those observations that are most similar to each other based on some selected measure of dissimilarity, referred to as a “linkage” (Friedman et al., 2001). The number of retained clusters can then be selected either using some measure of cluster (dis)similarity or a pre-selected value. We use agglomerative clustering, which additionally requires the selection of a method through which the dissimilarity of separate clusters is calculated. A full discussion on hierarchical clustering may be found in (Friedman et al., 2001). Herein, we chose the number of clusters to be identical to the number of principal components. Complete linkage was used to calculate the difference between clusters at each iteration. Single and average linkage displayed a propensity for resulting in clusters that were very large. This was most likely due to the fact that single linkage considers the minimum distance between clusters at each iteration, thus regarding clusters as more similar in general. Complete linkage considers the maximum distance between clusters and thus considers clusters to be more distinct. Average linkage is the average of these two extremes.

## 4 Results

**FIGURE 4:**
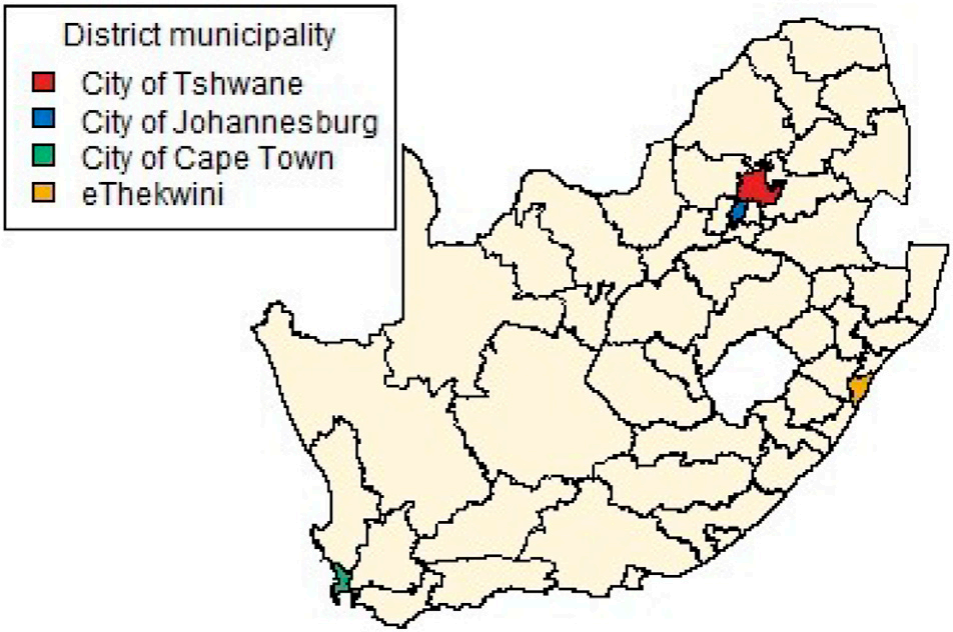
South Africa’s district municipality boundaries and locations of four largest cities.

Figure 4 shows the 52 district municipalities of South Africa. The four largest cities in the country are Tshwane, Johannesburg, Durban and Cape Town, situated in the City of Tshwane, City of Johannesburg, eThekwini and City of Cape Town district municipalities respectively as indicated in colour in Figure 4. These four cities are the focal point of economic activity and travel in the country, and it is thus logical that they would play a substantially larger role in the transmission of the virus than other municipalities.

### 4.1 Method 1: Distance Method

**FIGURE 5:**
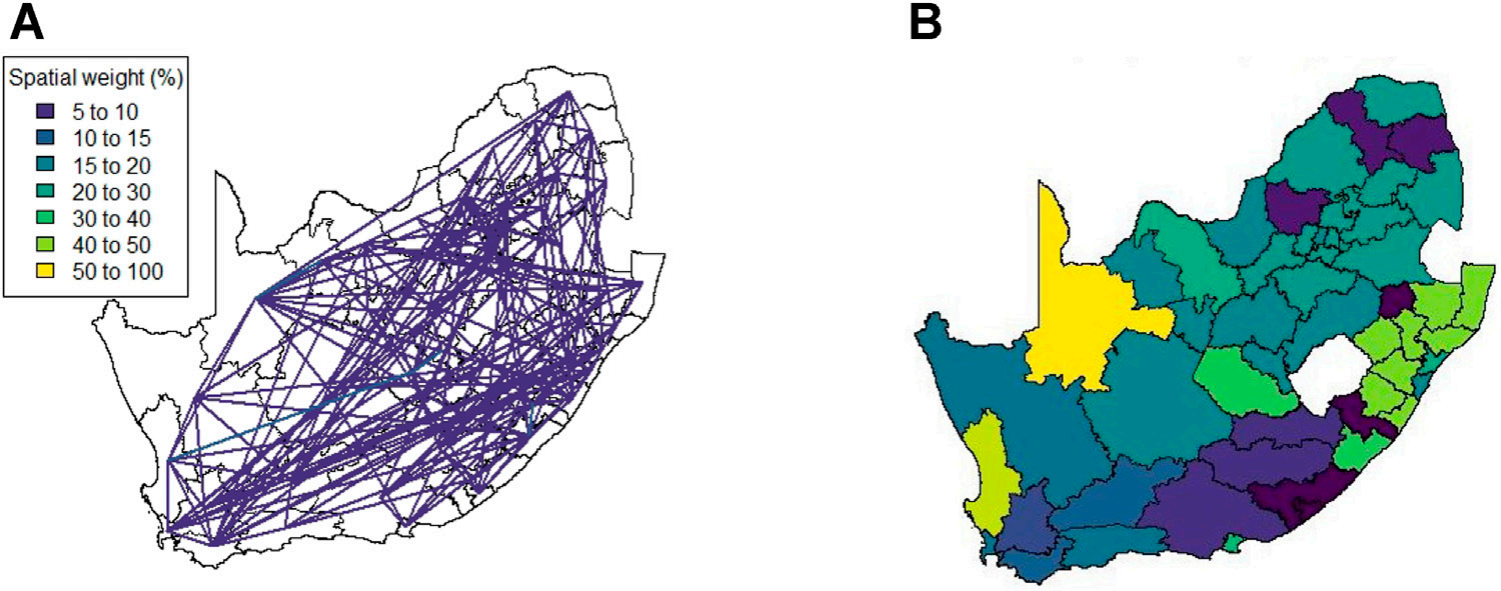
Method 1 **(A)** Spatial weights (weights ≤5 not shown), **(B)** Complete linkage clustering (14 clusters indicated by colours).

Figure 5A shows the weights (those >5) for the exponential distance weight matrix. Since the entries are calculated based only on the Euclidean distance between the district municipalities (and no additional information), there are no significantly large weights present. As temporal information cannot be included, this method produces only a single spatial weight matrix.

This spatial weight matrix required the largest number of principal components, namely 14, in order to explain 75% of the variation in the data. This is most likely due to the lack of any form of auxiliary data or information that could be used to better describe the relationship of the district municipalities. The result of hierarchical clustering on the principal component observations is given in Figure 5B.

### 4.2 Method 2: Mobile Network Method

**FIGURE 6:**
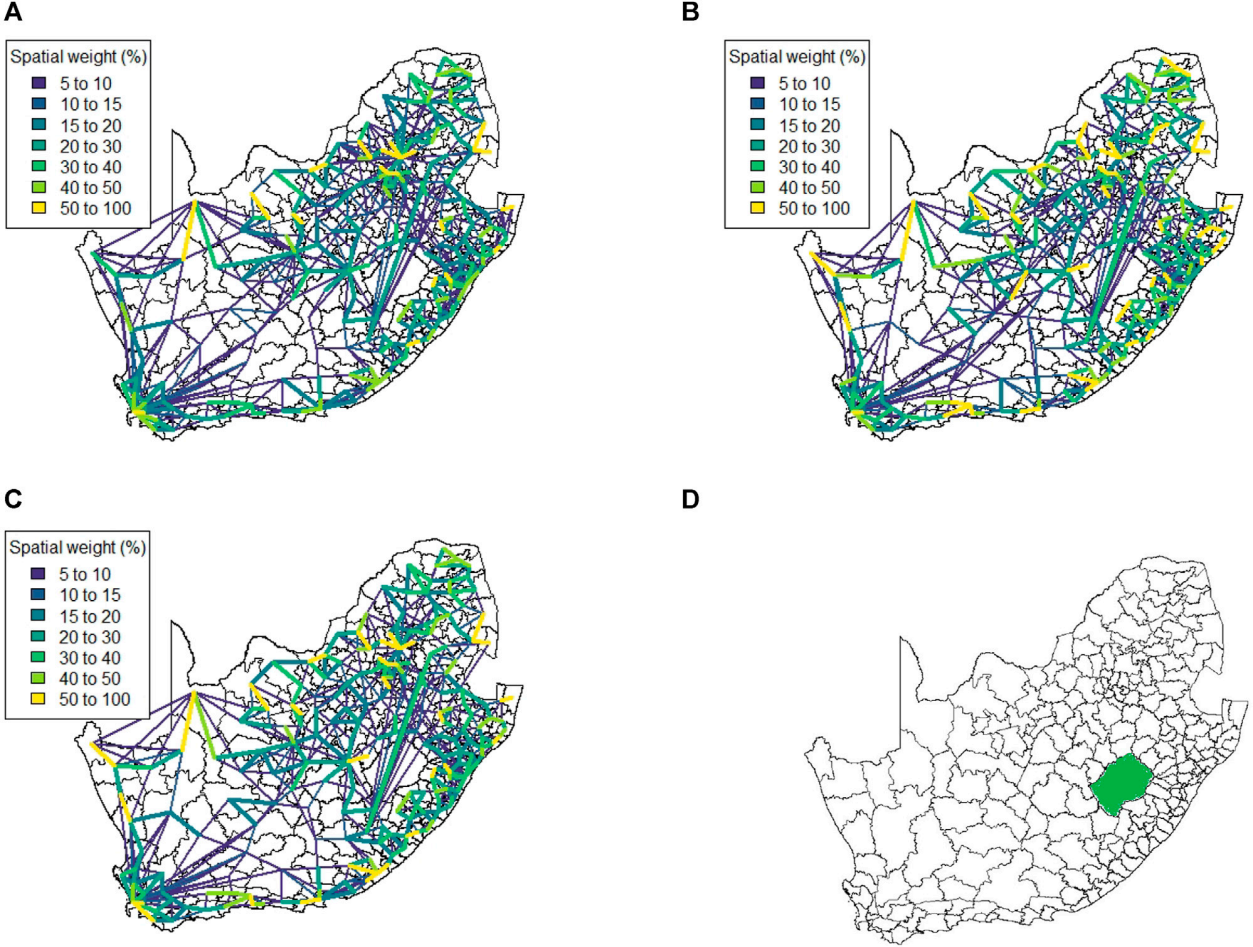
Method 2 spatial weight matrix entries (weights ≤5 not shown) **(A)** Business as usual, **(B)** Level 5, **(C)** Level 4, and **(D)** South Africa at local municipality level (neighboring country Lesotho in green).

Figure 6 shows the spatial weight matrix for every level of lockdown that the mobile phone data spans at administrative level 3. This spatial weight matrix identifies very strong spatial associations over relatively shorter distances (indicated by the yellow lines). These strong correlations appear to cluster around the edges of the country, with locations in the centre of the country displaying less spatial association overall.

**FIGURE 7:**
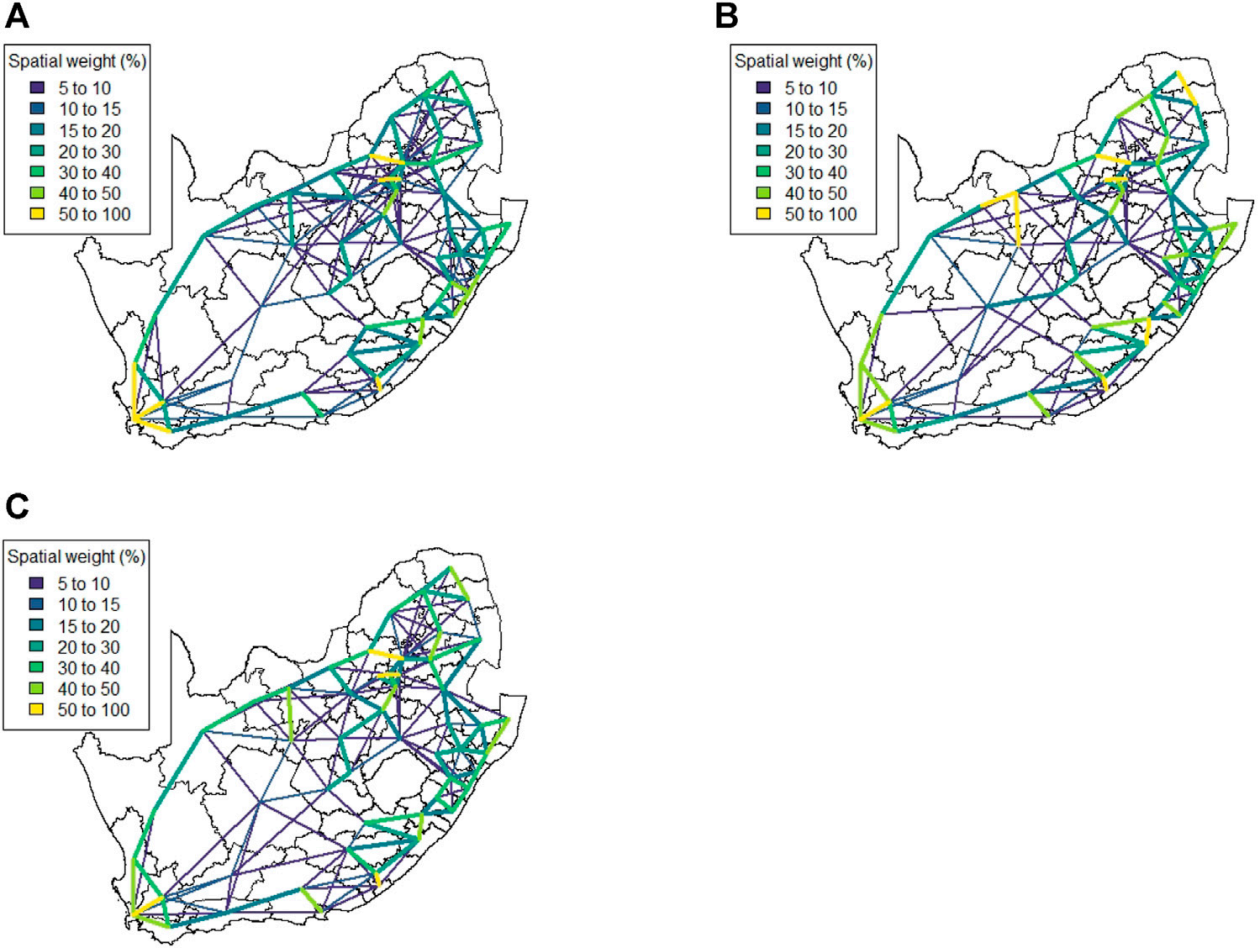
Method 2 spatial weight matrix entries (weights ≤5 not shown) **(A)** Business as usual, **(B)** Level 5, **(C)** Level 4 at district municipality level.

We note that there are strong spatial associations that do not appear to be associated with any of the four major cities in the country. In particular, we note strong associations in the North-Western region of the country as well as some spatial associations across Lesotho (a neighbouring country that is landlocked by South Africa, shown in Figure 6D). The spatial weight matrices for the mobile network data were also aggregated to administrative level 2, shown at Figure 7. While some strong spatial associations can still be identified around the country’s borders, many previously identified associations (including several significant associations spanning across the neighboring country of Lesotho) are now negligible. It is clear that while this lower spatial resolution does capture some of the spatial associations present in the data, much information is lost when aggregating between spatial resolutions.

**FIGURE 8:**
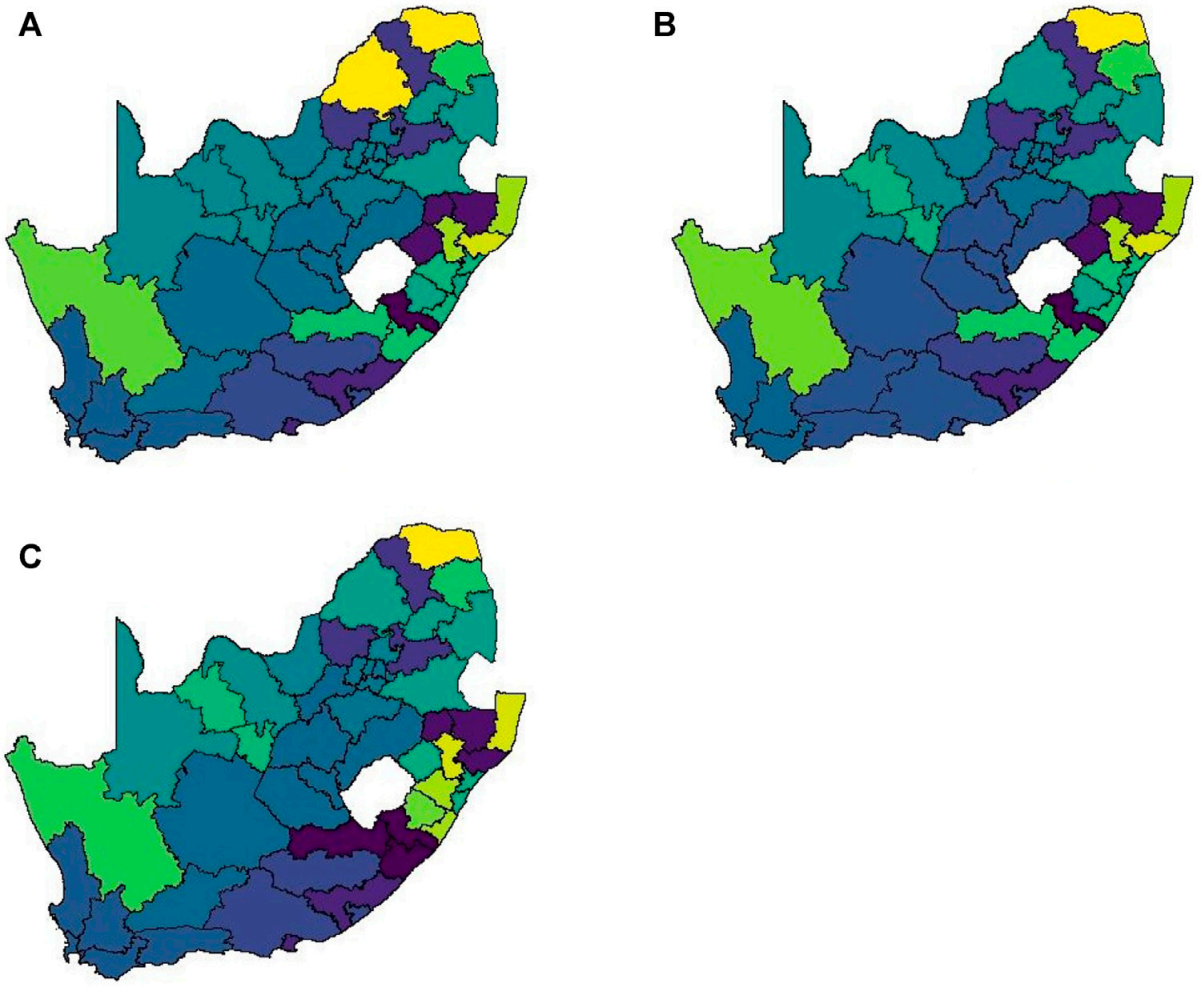
Method 2 complete linkage clustering results (20 clusters) **(A)** Business as usual, **(B)** Level 5 and **(C)** Level 4.

A notable drawback of data being at such a high spatial resolution is that it becomes very difficult to cluster locations in a meaningful way. At administrative level 3 there are 213 spatial units to consider. In order to explain just 75% of the variation in this data one requires approximately 70 principal components. Such a high number of clusters does not lend itself to easy interpretation and thus it is necessary to aggregate to a lower spatial resolution to render analysis feasible. When aggregating to administrative level 2 we find that 20 principal components are required to retain 75% of the variation present in the data. This is most likely due to the fact that the mobile network exhibits far greater daily variation than our data sources. Figure 8 shows the clustering solution.

### 4.3 Method 3: Weighted Facebook Data Method

**FIGURE 9:**
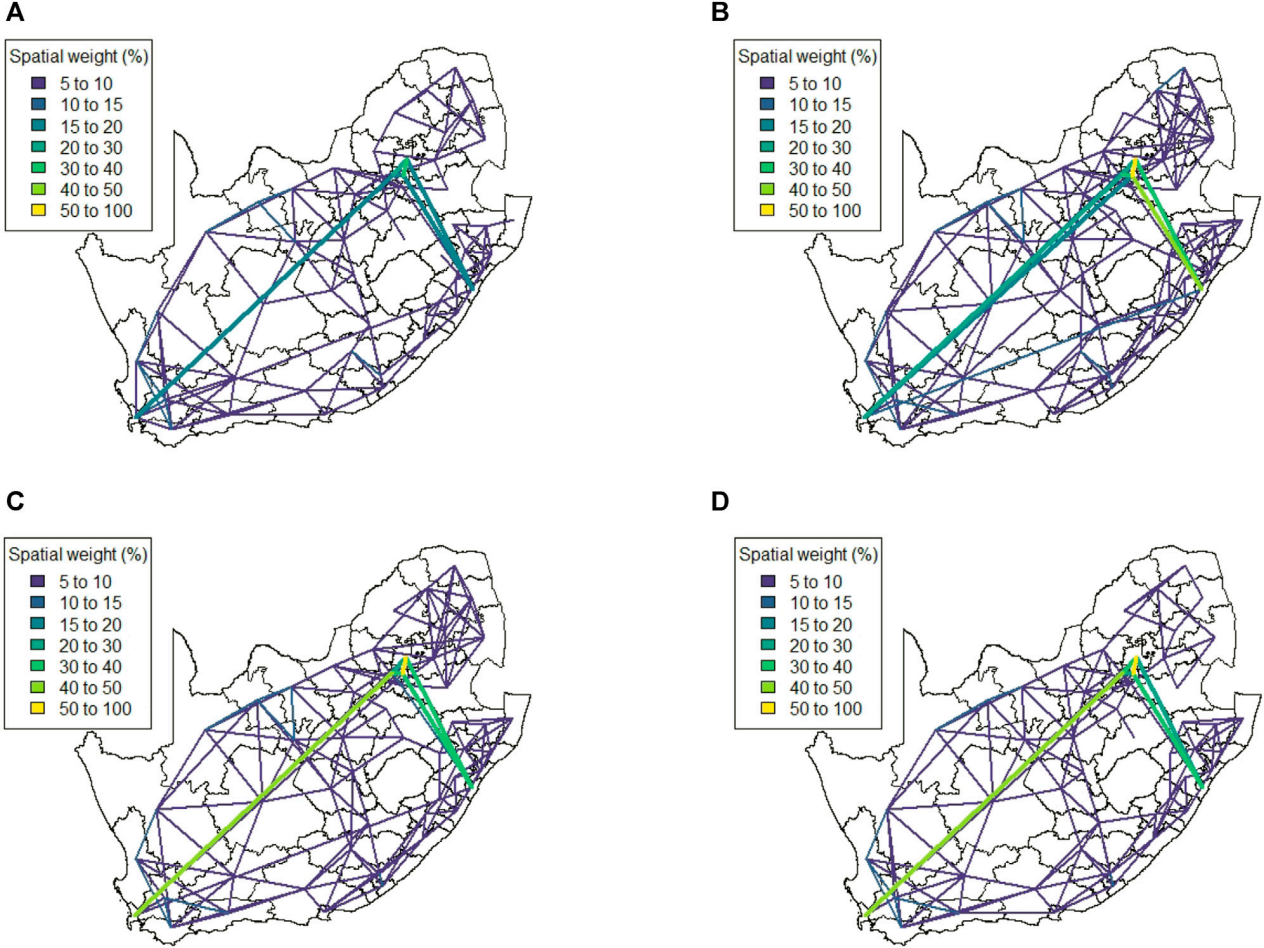
Method 3 spatial weight matrix entries (weights ≤5 not shown) **(A)** Business as usual, **(B)** Level 5, **(C)** Level 4, and **(D)** Level 3.

**FIGURE 10:**
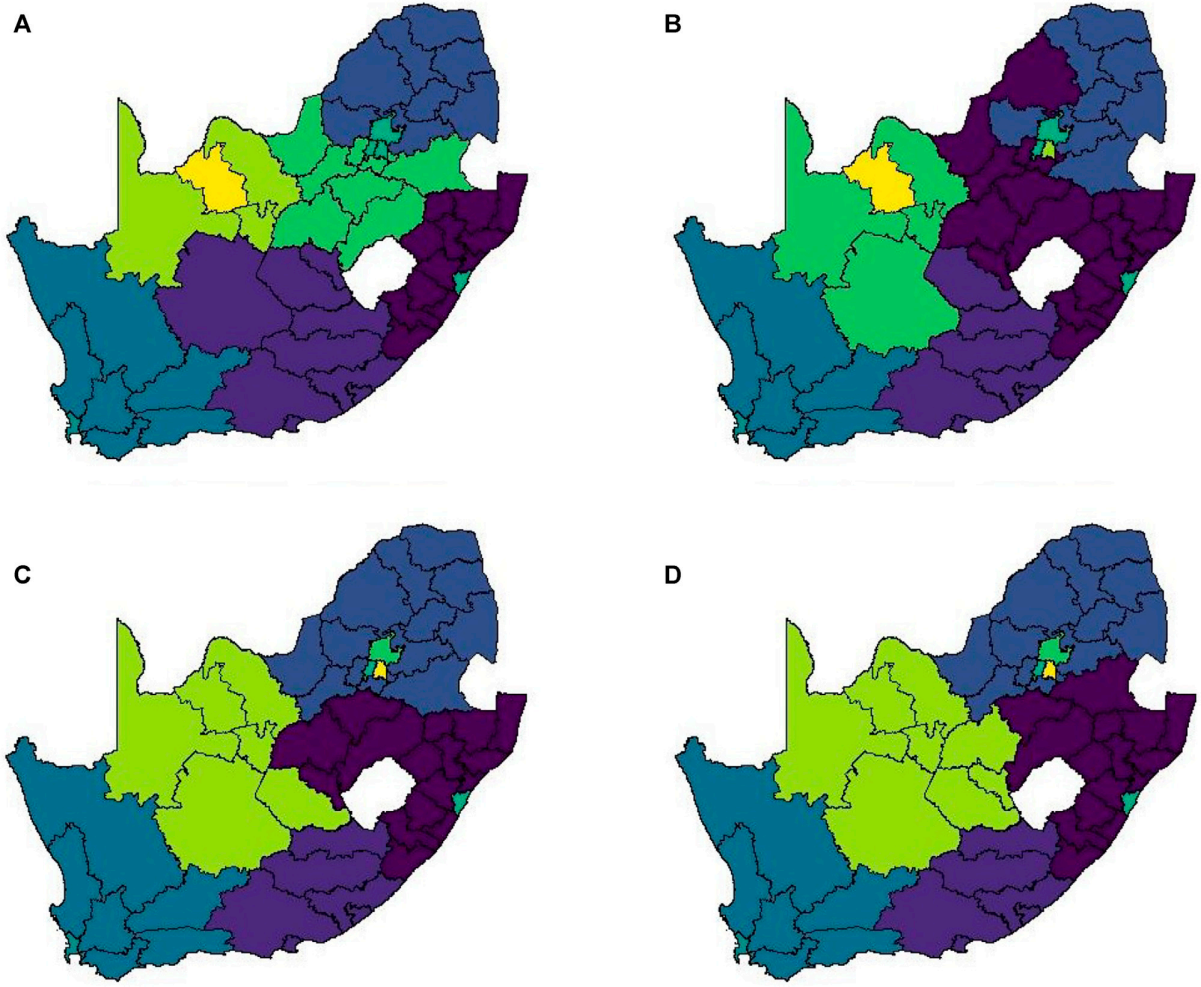
Method 3 complete linkage clustering results (9 clusters) **(A)** Business as usual, **(B)** Level 5, **(C)** Level 4, and **(D)** Level 3.

This matrix construction incorporates both the Facebook population mobility data and the population size for each district municipality into the spatial weights for each district municipality pair. Figure 9 shows the resulting matrix for each level of lockdown. By allowing both mobility and population size to play a role in this matrix, the strong spatial association between the four largest cities in South Africa is identified, despite the large geographical distance between them. If only Euclidean distance had been taken into account, this association would have been missed, as with Method 1. This spatial weight matrix required nine principal components to explain 75% of the variation in the data. Figure 10 shows the results of applying hierarchical clustering to the principal component observations.

### 4.4 Method 4: Scaled Facebook Data Method

**FIGURE 11:**
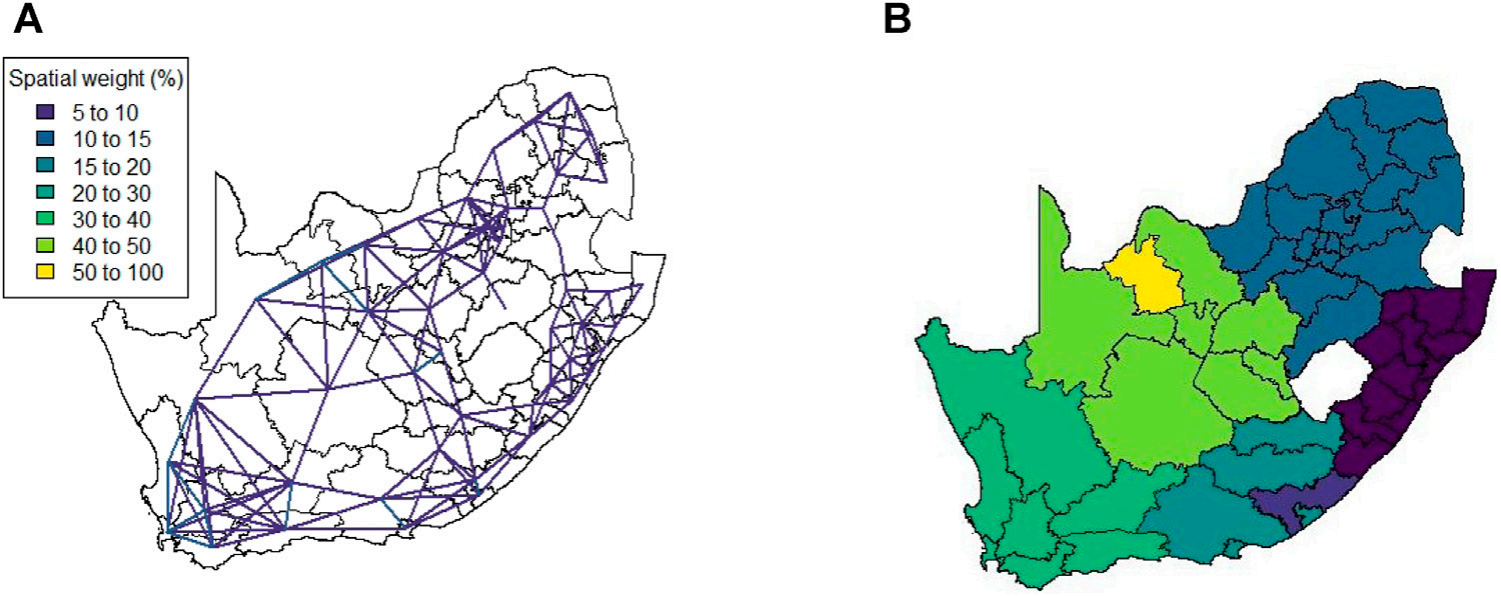
Method 4 **(A)** Spatial weights (weights ≤5 not shown), **(B)** Complete linkage clustering (7 clusters indicated by colours).

This spatial weight matrix was constructed as a potentially more realistic alternative to the exponential distance matrix. Despite containing a temporal element (in the form of daily mobility measurements retrieved from the Facebook data), the results for this matrix do not show any significant change across the various levels of lockdown. Figure 11 visualises the spatial weight matrix. Clustering performed on this matrix was more successful and intuitive. Only seven components were required to explain 75% of the variation in the data. Figure 11 shows the clustering solution.

## 5 Discussion

The results in Section 4 illustrate a number of ways to construct spatial weight matrices from mobility data. For the standard exponential distance method (Method 1), it is clear from Figure 5 that the clustering solution on this spatial weight matrix is not ideal. There are far too many clusters and the clustering solution reveals no clear interpretation. Although the initial matrix construction used only the distances between district municipalities, district municipalities that were located closer together were not generally clustered together. The entries of the spatial weight matrix constructed using the mobile network data (Method 2), shown in Figures 6, 7, reveal strong spatial associations over relatively short distances. The four focal largest cities in the country are clearly identified as hubs for high mobility but there are other regions, particularly those situated on or near the borders of the country, that showcase highly concentrated mobility. A possible explanation for these strong spatial associations being observed far away from cities is the existence of mining activity in these areas. Given that South Africa has a very large and widespread mining sector, it seems only reasonable that any model with a spatial element should strive to incorporate these associations. The clustering solution for this spatial weight matrix, shown in Figure 8, is distinct from the other solutions in this paper in that distance is clearly not a key role player in deciding which spatial units are clustered together. Many spatial units that are situated close to one another in geographical space are not clustered together, and some spatial units are even placed into their own clusters despite having many spatial neighbours. It can be argued that this clustering solution is a more realistic reflection of the amount of travel between spatial units. The reason for this is that locations being situated closer together does not always imply that there is a higher degree of travel between these locations. The strong local connectivities picked up by this method are useful for epidemiological modelling, for example, prediction of case number hotspot movement into spatial units of higher likelihood of mobility. The four largest cities in South Africa are Tshwane, Johannesburg, Cape Town and Durban, situated in the City of Tshwane, City of Johannesburg, eThekwini and City of Cape Town district municipalities respectively, as shown in Figure 4. The results in Figure 9 (method 3) show a large spatial association between these locations prior to the implementation of level 5 lockdown. Under level 5 restrictions, when the spatial influence of most district municipalities decreased, the spatial influence between these four locations became more pronounced by comparison. This most likely indicates that while smaller district municipalities were less active due to restrictions, these four were comparatively more active and still saw a sizable amount of travel between them. This seems feasible, given that these locations are the focal points for economic activity in the country and thus could not reasonably become “immobile”. As restrictions were lifted, these spatial weights were still significantly larger than those for other district municipalities, indicating that, despite restrictions being eased, the spatial influence between these four places is still significantly stronger than before the lockdown. It is also apparent that the spatial influence between less influential district municipalities has not returned to the level that they were during business as usual (pre-lockdown). Figure 10 shows that the district municipalities housing the four largest cities are all either clustered together or in clusters of their own. Other district municipalities are generally clustered together based on the distance between them. This clustering solution indicates that the four largest cities are significantly different from the locations around them. This spatial weight matrix is thus able to pinpoint the fact that these locations play a potentially larger role in spatially-dependent phenomena such as the spread of a virus. The effect in epidemiological modelling allows for longer range spatial dependency, for example, spread of the virus by daily flights between major city hubs. This is not captured by Method 2. The clustering results for Method 4, shown in Figure 11, do not display any significant changes over the various levels of lockdown. Figure 11 also shows that the clusters that are formed for this spatial weight matrix are clearly based primarily on distance, but illustrates that the auxiliary Facebook data aids in constructing more finite and sensible clusters. Interestingly, we notice a district municipality that has been classified into a cluster on its own. When inspecting the results for the other spatial weight matrices we note that this district municipality has previously also been identified as its own cluster and was shown to have strong spatial associations for Method 2. Upon further inspection we note this district municipality houses several mines. Similarly to Method 2, this spatial weight matrix is able to identify location associations that go unnoticed when relying on simple concepts such as Euclidean distance. This method may not be useful alone in epidemiological modelling and should most likely be used in conjunction with either Method 2 or 3. This paper shows that different representations of spatial data can offer a variety of insights and capture different relationships in the data. For example, the spatial weight matrix created using Method three data emphasises the prominent role of focal points in population activity. However, the spatial weight matrix constructed using Method four offers a scaled and smoothed way to use distance to indicate which locations have a higher spatial influence on one another. These two spatial weight matrices use the same spatial data (i.e. the Facebook for good data), but offer vastly different interpretations of spatial influence. Finally, the interpretations that were able to be made from the mobile phone data indicates that there are many potentially strong spatial associations at shorter distances that can only be identified when inspecting data at a high spatial resolution. Table 4 provides a summary of the methods used in this paper, their strengths and weaknesses, and their usability based on the results. Each of these representations can be seen as valid and are complementary with regards to the insight they offer. Depending on the specific phenomenon under study, an argument could be made their usability based on observed patterns from the results, as in the case of a pandemic such as COVID-19, which affects not only congregated communities but allows for consequences to be felt across an entire country.

**TABLE 4:** Spatial weight matrices comparison.

*Spatial weight matrix*	*Pro*	*Con*	*Interpretation/Contribution*
Method 1 - Distance	Simple to construct and understand Used often in literature	Less realistic	Convenient to use and easy to understand and interpret. Not realistic enough for real insight
		Inadequate for clustering	
		Lacks temporal element	
Method 2 - Mobile network	High spatial resolution Large amounts generated passively by mobile device users	Computationally expensive	Captures strong spatial associations over relatively short distances. Allows for the identification of patterns potentially missed by other methods
		Difficult to obtain	
		Not representative	
		Privacy concerns	
Method 3 - Weighted Facebook data	Freely available data Potentially more representative	Low spatial resolution	Captures association between focal points of human activity regardless of distance
		Lacks specificity	
Method 4 - Scaled Facebook data	Simple to construct and understand. Freely available data Potentially more representative	Lacks temporal elements	Adds additional information to previously simplistic model. Additional information improves clustering
		Low spatial resolution	

Understanding mobility during the current pandemic is essential. Both the reduction in mobility as well as retained mobility need to be well understood, and depend on reliable data collection. As shown here, data are collected in different ways and are also made available in a variety of formats. Mobility is distributionally different across strata of a region’s demographics, with more mobile locations likely to result in higher disease transmission. Higher resolution mobility data is important to capture these differences in more detail. Even so, the spatial resolution at district municipality captures these nuances of the movement under each lockdown level, and shows that significant movement still took place due to the vulnerability of a large portion of South Africa’s population.

The possibility of micro-spatial estimation (small area estimation) is something to investigate further. Making use of demographic covariates, transport networks and as well as mobile network coverage maps could provide connectivity matrices at higher spatial resolution, ideally at ward level. Estimation at higher spatial resolution could be done by making use of a number of lower spatial resolution sources. This allows for micro-scale modelling of COVID-19 spread and will allow for privacy while increasing spatial resolution and providing deeper coverage in a region. Google mobility data is also available^8^ but only at provincial level (administration level 1) for South Africa. This spatial resolution is too low to consider estimation down to ward level, especially if alternative mobility data is available at administrative level 2. However, one could also combine mobility data at different spatial resolutions in a way that takes advantage of the strengths of each dataset.

The computational aspects of dealing with mobility data should not be overlooked. Spatial weight matrices can become very large, depending on the number of spatial regions under consideration. Herein the matrices were not sparse, meaning that sparse representations could not be used. Sparse representations could be investigated for high spatial resolution modelling.

To quantify the similarity between the different spatial weight matrices, one might consider the use of simple parametric measures of correlation such as Pearson’s correlation coefficient. However, given that there are a total of 52 spatial units (at a district municipality level) and the weights between many spatial unit pairs are negligible, the spatial weight matrices can be regarded as zero-inflated. In addition to making no allowance for the spatial nature of the data, namely the spatial dependency, standard measures of correlation would also deliver biased results. Future research could investigate methods for comparison of spatial weight matrices via appropriate correlation calculations or other techniques.

## 6 Conclusion

COVID-19 spreads spatially and thus the importance of mobility data for COVID-19 modeling should not be disregarded. Ideally, the raw data from the mobile network providers and Facebook, if available, could provide individual movements, allowing for accurate construction of spatial weight matrices. This data could be anonymised and shared. However, instead the methods proposed here can be made use of. The use of movement data in epidemiology is becoming an important covariate to include, without which the spread can only be modelled in isolated regions. Social interactions between human beings are unavoidable. Simple spatial weight matrix construction techniques, such as only taking into account distances, are not always ideal when the spatial associations being captured are dependent on covariates which are not only proximity based. This is made clear by the observed poor performance of Method 1 when it was used as the basis of clustering. The methods presented herein and the results shown also enable epidemiological modellers in considering how to incorporate spatial relationships in models. This is seldom done due to limited mobility information as well as modelling complexities it introduces. However, the improved accuracy in model outcomes will ultimately balance out computational complexities. The paper provides insights into mobility data availability, representability as well as construction for use in spatial modelling. Future research should investigate estimation to a higher spatial resolution using multiple data sources as well as the effect of spatial resolution in spatial epidemiological modelling.

## Data Availability

The data analyzed in this study is subject to the following licenses/restrictions: The mobile network data used in this study is not directly available without approval, so cannot be shared directly with the paper. Requests to access these datasets should be directed to https://research.fb.com/blog/2020/06/protecting-privacy-in-facebook-mobility-data-during-the-covid-19-response.

## Footnotes

1
https://povertydata.worldbank.org/Poverty/Home (Accessed May 2021)

2
https://dataforgood.fb.com/(Accessed May 2021)

3
https://docs.microsoft.com/en-us/bingmaps/articles/bing-maps-tile-system (Accessed May 2021)

4 Mid-2021 Statistics South Africa Population Report http://www.statssa.gov.za/publications/P0302/P03022021.pdf (Accessed August 2021)

5 See https://www.geopoll.com/blog/mobile-penetration-south-africa/and https://www.icasa.org.za/uploads/files/State-of-the-ICT-Sector-Report-March-2020.pdf (Accessed May 2021)

6 Mid-2021 Statistics South Africa Population Report http://www.statssa.gov.za/publications/P0302/P03022021.pdf (Accessed August 2021)

7 All analysis presented here was performed on a desktop computer running Intel Core i7 with a clock speed of 3.40GHz, a 64-bit operating system and 64 GB of installed memory

8
https://www.google.com/covid19/mobility/(Accessed May 2021)

## Appendix

Facebook for good data calculation.

Let *u* represent a single individual and *U*_*t*_,_*i*_ represent district municipality *i* at time *t*. The total number of Bing tiles visited by inhabitants of district municipality *i* is then

$$total\text{\_}\text{tiles}\left(U_{t,i}\right)=\underset{u\in U_{t,i}}{\sum }\min\left(tiles\left(u\right),200\right).$$

Note that the maximum number of Bing tiles visited that a single individual can contribute is restricted to 200. In order to preserve user privacy, an error term was included by drawing from a Laplace distribution with parameters 0 and $\frac{F}{\epsilon }$ where *F* = sensitivity parameter and ϵ = noise parameter as follows

$$total\text{\_}{\text{tiles}}^{\prime }\left(U_{t,i}\right)=total\text{\_}\text{tiles}\left(U_{t,i}\right)+Laplace\left(0,\frac{F}{\epsilon }\right).$$

The average number of tiles per district municipality was then calculated as

$$avg\text{\_}{\text{tiles}}^{\prime }\left(U_{t,i}\right)=\frac{total\text{\_}{\text{tiles}}^{\prime }\left(U_{t,i}\right)}{\left|U_{t,i}\right|}.$$

The mobility value for each district municipality and for each day was then finally expressed with respect to the baseline as

$$F_{i}^{\left(t\right)}=\frac{avg\text{\_}\text{tiles}\left(U_{t,i}\right)-baseline\text{\_}\text{avg}\text{\_}{\text{tiles}}^{\prime }\left(i,day\text{\_}\text{of}\text{\_}\text{the}\text{\_}\text{week}\left(t\right)\right)}{baseline\text{\_}\text{avg}\text{\_}{\text{tiles}}^{\prime }\left(i,day\text{\_}\text{of}\text{\_}\text{the}\text{\_}\text{week}\left(t\right)\right)}.$$

For further details regarding this data see https://research.fb.com/blog/2020/06/protecting-privacy-in-facebook-mobility-data-during-the-covid-19-response/.
